# From desk to bed: Computational simulations provide indication for rheumatoid arthritis clinical trials

**DOI:** 10.1186/1752-0509-7-10

**Published:** 2013-01-22

**Authors:** Jennifer E Dent, Christine Nardini

**Affiliations:** 1 Group of Clinical Genomic Networks, Key Laboratory of Computational Biology, CAS-MPG Partner Institute for Computational Biology, Shanghai Institutes for Biological Sciences, Chinese Academy of Sciences, Shanghai, PR China; 2 Population Health Research Centre, Division of Population Health Sciences and Education, St. George’s University of London, Cranmer Terrace, London, UK

**Keywords:** Rheumatoid arthritis, Tyrosine kynase, Simulation modelling, BioLayout express

## Abstract

**Background:**

Rheumatoid arthritis (RA) is among the most common human systemic autoimmune diseases, affecting approximately 1% of the population worldwide. To date, there is no cure for the disease and current treatments show undesirable side effects. As the disease affects a growing number of individuals, and during their working age, the gathering of all information able to improve therapies -by understanding their and the disease mechanisms of action- represents an important area of research, benefiting not only patients but also societies. In this direction, network analysis methods have been used in previous work to further our understanding of this complex disease, leading to the identification of CRKL as a potential drug target for treatment of RA. Here, we use computational methods to expand on this work, testing the hypothesis *in silico*.

**Results:**

Analysis of the CRKL network -available at http://www.picb.ac.cn/ClinicalGenomicNTW/software.html- allows for investigation of the potential effect of perturbing genes of interest. Within the group of genes that are significantly affected by simulated perturbation of CRKL, we are lead to further investigate the importance of PXN. Our results allow us to (1) refine the hypothesis on CRKL as a novel drug target (2) indicate potential causes of side effects in on-going trials and (3) importantly, provide recommendations with impact on on-going clinical studies.

**Conclusions:**

Based on a virtual network that collects and connects a large number of the molecules known to be involved in a disease, one can simulate the effects of controlling molecules, allowing for the observation of how this affects the rest of the network. This is important to mimic the effect of a drug, but also to be aware of -and possibly control- its side effects. Using this approach in RA research we have been able to contribute to the field by suggesting molecules to be targeted in new therapies and more importantly, to warrant efficacy, to hypothesise novel recommendations on existing drugs currently under test.

## Background

Rheumatoid Arthritis (RA) is a complex disease involving a yet unknown number of molecules and their alterations (from susceptibility genes [1], to unsustained methylation [2], to metagenomic alterations [3]). The disease affects a large number of organs, tissues and sites across the body [4-7], typically causing recruitment and activation of inflammatory cells, synovial hyperplasia and destruction of cartilage and bone. A complete loss of mobility and functioning can be the final evolution of the disease [8]. Affecting approximately 1% of the population worldwide [9], extensive research into the treatment of the disease is thus warranted. Highly relevant to RA research is the identification of new therapies as, in fact, some of the most common drugs used to treat RA, such as MTX (Methotrexate [10], the most common Disease Modifying Antirheumatic Drug, DMARD) can cause further liver, lung and kidney damage as well as strong immunodepression. More advanced therapies that target focused pathways (anti-*TNF*α [10]) and reduce -but do not eliminate- this type of side effect are extremely costly, whereas traditional approaches receive controversial favour and lack molecular evidence [11].

To gain more insight into the basic mechanisms of action of the disease and to develop more specific and useful drugs, molecular data have been collected and combined so that the whole structure of the molecular networks involved in RA can be studied [12]. Specifically, gene microarray data have contributed greatly to pathogenesis and to the identification of biomarkers for diagnosis, to patient stratification and prognostication of RA [12]. Other methods such as Genome-Wide Association Studies (GWAS) have been used to scan the whole genome in search of loci susceptible to carry mutations related to RA [13-15] and in some cases information from these 2 approaches are joined to better predict candidate susceptibility genes of the disease [16]. Furthermore, some signal transduction pathways have also been identified as being involved in the disease and have been recommended as drug targets to treat RA [17-19].

Where possible, these data have already been combined and analysed in [12], in which a comprehensive map for RA was built to combine together the molecules and pathways that were so far found to be associated with RA, based on systemic, high-throughput data and made available following the *CellDesigner* standard [20] (see also Additional File 1). Expanding on this work, here we present the outcome of further investigation into one of the most important results from the aforementioned publication. Specifically, we investigate further the potential of CRKL (approved name: v-crk sarcoma virus CT10 oncogene homolog (avian)-like), and its close network, as a drug target for RA. CRKL is believed to activate a number of signalling pathways and may also be involved in tumour growth. As CRKL is currently not a known drug target for RA, it is potentially interesting for further research [21].

In the present study, the hypothesis that CRKL could act as a potential drug target has been tested using computational methods to simulate the likely effect that perturbing CRKL will have on the rest of the molecular interaction network presented in [12]. In order to achieve this, the up- and down-regulation of CRKL have been simulated using the computational software *BioLayout Express*[12] and a biological interpretation of the results is discussed. By adding here *dynamics* and regulations to a sub-network of the *static* reconstruction of the global molecular network in [12], the current study allows us to show how, in time, perturbation of the expression of molecules of interest in the network can lead to a novel state of up- or down- regulation of other molecules in the network.

## Methods

### Network reconstruction

In the first instance, a directional network of molecular interactions between components involved in RA, including CRKL, was extracted from the RA map publically available at [22]. Where necessary, Disease Atlas [23] was used to clarify or disambiguate the literature regarding the known state of the maps’ molecules in RA.

As the original map contained some nodes that were not connected to CRKL (the molecule of interest in this study), the aforementioned *static interaction map* was trimmed using Cytoscape [24]. Specifically, using Tarjan’s algorithm, available in the Cytoscape plug-in BiNoM [25], all nodes that were strongly connected to CRKL were identified, forming the core of the CRKL sub-network. Nodes that were weakly connected to the CRKL core-network (i.e. those nodes that are connected to CRKL in only one direction) were identified and added back to the network using a clustering algorithm in Cytoscape that considers node overlap [25]. In this way, isolated clusters of nodes (isolated clusters have no effect on the rest of the network) and pathways that were part of the original RA map but did not contain CRKL and/or could not be affected by perturbation of CRKL (due to limitations in connectivity between pathways) were removed. The resulting network, shown in Additional File 2 (and available for download in *graphml* format at [26]) contains 223 molecules linked to CRKL. In order to use the pathway diagram as a resource for modelling the CRKL network, and to simulate a dynamic element, the Signalling Petri Net (SPN) algorithm proposed in [27] was adopted.

### (Signalling) Petri Net

Petri net models, first described in 1939 [28], characterise the dynamics of signal flow using token distribution and sampling. Specifically, a Petri net is a directed network in which nodes are connected by transitions, where the edges describe the conditions for which transitions can occur. Nodes in a Petri net contain a discrete number of ‘tokens’, the distribution of which across all nodes describes the state of the system. In a Petri net a transition causes the number of tokens at a node to change by ‘firing’ whenever there are sufficient tokens at the starting node of an edge. When a transition fires, tokens are placed at the end node of the edge over which the transition occurs. The execution of a Petri net is nondeterministic so that when multiple transitions are enabled at the same time any one (or none) of them may fire, thus representing the stochastic nature of the system.

Signalling Petri net (SPN) extends the Petri net model by allowing for nonparametric modelling of cellular signalling networks; adding a simulator for modelling the average flow of tokens over multiple time steps [27]. Compared to Petri nets, SPN can model different transitions and different node types, corresponding to those available in the commonly used System Biology Mark-up Language (SBML). SBML is “a machine-readable format for representing models, oriented towards describing systems where biological entities are involved in, and modified by, processes that occur over time” [29]. SBML is particularly suitable for representing models commonly found in research on cell signalling pathways, metabolic pathways, biochemical reactions and gene regulation [29] to give examples, thus making it an appropriate language to adopt here. A major advantage of using software based on SBML is that it allows the systems biology community to share, evaluate and cooperatively develop models.

The addition of a simulator in SPN allows for one to repeat the process of *firing tokens* over multiple time blocks and to determine the state of a system on average and after perturbation. Here, using BioLayout Express, a software for the visualisation of biological data as networks [30] that incorporates SPN, it was possible to allow each node of the CRKL sub-network to represent a molecule and the number of tokens associated with a node at each time point to represent its expression level.

### Network simulation

By altering the number of tokens associated with CRKL at time *t = 0*, BioLayout Express was used to simulate a change in expression level of CRKL and the potential consequential effect on the CRKL sub-network. To achieve this, the CRKL sub-network was first transformed by adding transition gates to the network. Nodes at the edge of the network -representing the beginning of a path- were (arbitrarily) assigned 100 tokens at time zero. Those nodes that were at the end of a path were allowed to lose a random number of tokens at each time point chosen, as with movement of tokens downstream, uniformly from 0 to the number of tokens present at the parent node. This is necessary to avoid a build-up of tokens at the end of a pathway as this would lead to biased results for nodes that have zero out-degree (i.e. nodes from which no other node can be reached). Biologically, this assumption represents self-regulation of molecules. To simulate up- and down-regulation the number of starting tokens for CRKL was increased to 500 and reduced to 10, respectively. Each scenario (control, down-regulation and up-regulation, with 100, 10 and 500 tokens at time zero, respectively) was simulated 500 times, over 20 time points. The mean number of tokens per node per time point was recorded (mean calculated over all simulations, 500, per scenario).

### Interpretation

The t-value and corresponding p-values were calculated for each node in its *active* state (*n.b.* the original static network distinguishes between molecules in their active and inactive state to allow, for example, other proteins to catalyse activation). Namely, for each of the 223 nodes of the network the statistical significance of the 20th, and thus most stable, time point was computed by calculating the t-value for the control and perturbed distributions according to the equation:

$t-\mathrm{value}=\frac{\mu_{i,c}-\mu_{i,p}}{\sqrt{\frac{\sigma_{i,c}^{2}}{500}+\frac{\sigma_{i,p}^{2}}{500}}}$, where the *μ*_*i*_ ' *s* and *σ*_*i*_^2^ ' *s* represent the mean and variance of the number of tokens at node *i* at time point 20, for the control (c) and perturbed (p) networks. Comparing the absolute t-value with the corresponding critical value (t_0.05, 500_ = 1.965), those molecules whose expression level significantly changed after perturbation of CRKL were identified.

After obtaining the novel equilibrium state of the system, to verify whether or not the control on the mechanisms of interest was already under way via existing drugs (and to investigate the possibility to translate them to RA), we started our query from the *Drugs and Compound* overlapping for relevant molecules, using the GeneCards human gene database [31,32], returning to the literature and on-going studies for further supporting material. This discussion is turned to the RA-relevant case i.e. the forced down-regulation of CRKL as a possible therapeutic approach as, in fact, CRKL appears to be up-regulated in the disease.

Following the public availability of the RA network used to inform this study, the network analysed here is also made publically available at [26], where is can be saved as a *graphml* file, suitable for running SPN in BioLayout Express.

## Results and discussion

**Figure 1:**
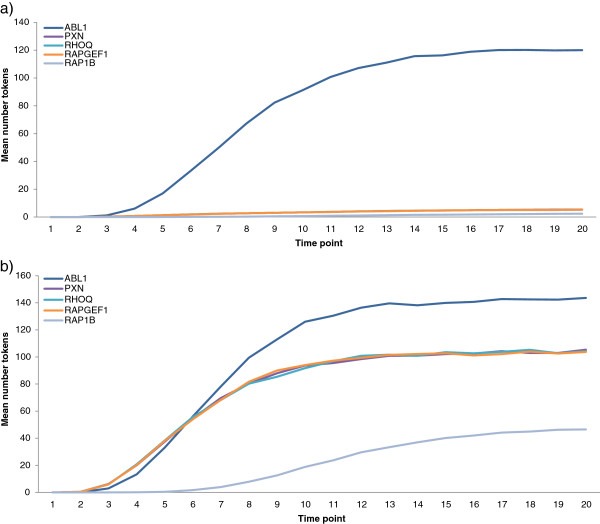
**Mean expression level of network molecules significantly affected by regulation of CRKL. a)** Simulated change in expression levels of molecules connected to CRKL, for CRKL down-regulated and **b)** Simulated change in expression levels of molecules connected to CRKL, for CRKL up-regulated. CRKL omitted (see Additional File 5 for CRKL included). Nodes correspond to those in Table 1.

For both scenarios (up- and down-regulation of CRKL), there was little difference in the manner in which expression levels of molecules changed over the twenty time points, with both scenarios resulting in two distinct groups of profiles: namely those that reach a high expression level in a short number of time points and a small number of less consistent more scattered profiles (see Additional File 3 and Additional File 4, which show examples of the expression profiles for molecules after up- and down-regulation of CRKL). In both scenarios, the majority (~76%) of molecules appeared to reach a stable threshold in the first 10 time steps. This result implies that when a single node is perturbed, the topological structure of the network as a whole remains stable, which is typical of biological networks [33]. Figure 1 (and Additional File 5) shows the expression profile patterns of the molecules that are significantly affected perturbation of CRKL, namely ABL1 (by CRKL up-regulation only), PXN, RHOQ, RAPGEF1 and RAP1B (full descriptions given in Additional File 6 - see also Table 1 and Discussion). These graphs are useful to observe the *dynamics* of the response as they indicate how the number of tokens builds up over time. However, these numbers are always positive and relative to the starting number of tokens at CRKL. Therefore, to determine if these differences in expression profiles are important, the change in the mean number of tokens per node at the 20th time point, compared to the baseline (here 100 tokens at time zero), was considered for CRKL up- (500 tokens at time zero) or down- (10 tokens at time zero) regulated. This enables one to further understand the affect that perturbing CRKL has on its neighbours. These differences are summarised in columns 5 and 6 of Table 1, which shows the most statistically significantly changed nodes.

**Table 1:** **Results of*t*-test comparing mean expression levels (at time point 20) for all scenarios**

**Node name**	**Mean number of tokens at 20th time point**			**Absolute t-value (p-value)**	
	**Control**	**CRKL up-regulated**	**CRKL down-regulated**	**CRKL up-regulated**	**CRKL down-regulated**
ABL1	130.05	145.27	120.51	2.51 (0.012)	1.7 (0.089)
CRKL	149.84	676.62	14.95	34.57 (<0.001)	39.34 (<0.001)
PXN	34.69	101.84	5.09	16.34 (<0.001)	20.54 (<0.001)
RAP1B	17.58	48.25	2.45	11.80 (<0.001)	14.96 (<0.001)
RAPGEF1	38.70	112.44	6.05	16.14 (<0.001)	19.34 (<0.001)
RHOQ	36.04	103.40	5.35	15.83 (<0.001)	19.10 (<0.001)

Knowing that down-regulation of CRKL is of interest, as the gene appears to be up-regulated in RA (see [34] and related articles), few words are necessary to discuss the case of ABL1, which was shown to be statistically significant only when CRKL was up-regulated: ABL1 is a well-known part of the network of interactions of CRKL, with its activity contributing to the phosphorylation -and therefore activation- of CRKL. The computational result indicating that ABL1 appears to be significantly affected by the enhanced activity of CRKL, but not by the reverse, is clearly interpretable in biological terms as ABL1 is, in a physiological condition, a trigger of CRKL activity. Therefore, CRKL activation is necessarily due to phosphorylation, requiring ABL1 to display significant levels of activity (CRKL up, ABL1 up). Conversely in our scenario, inhibition of CRKL is forced from outside the network with the token depletion that mimics the possible inhibitory activity of a novel drug. If this were to be done directly on CRKL, indeed inhibition of ABL1 would not be relevant (CRKL down, ABL1 free).

However, despite the smooth (biological) interpretation of this particular result, we wish to warn potential users of our approach in that such situations (one-sided statistical significance) can, in some cases, be ambiguous and possibly interpretable as unreliable (ABL1 is indeed no longer statistically significant at the 1% level for CRKL up-regulated). As always, computational results, obtained by more or less simplified modelling of the biological reality, require manually curated biological interpretation.

For CRKL down-regulated (desirable effect for the control of RA), we investigate the following further: RAP1B, RAPGEF1, PXN and RHOQ. The known interactions among these four molecules indicate that PXN phosphorylation activates CRKL, which triggers the RAS and JUN pathways involved in cytoskeleton remodelling and cell adhesion [21]. Considering first the central molecule in this sub-network (RAPGEF1), we observed that no compound was listed in [35] as being able to target the gene of interest. Since RHOQ and RAP1B are directly connected to RAPGEF1 in the activation of GTPases (hydrolase enzymes that can bind and hydrolyse guanosine triphosphate, GTP) we concentrated our efforts around PXN and CRKL to identify overlapping compounds for discussion.

CRKL is associated with a number of pathways, for instance the MAPK signalling pathway, chronic myeloid leukaemia and regulation of the actin cytoskeleton pathway. Most of these pathways are related to immune and inflammation reactions. However, as far as searching Pharmacogenomics Knowledge Base website [36] we found no evidence to suggest that CRKL is being used as a drug target. In the following we therefore discuss the implication of CRKL down-regulation only, as this is relevant to our original goal of identifying CRKL as possible drug target in RA.

CRKL is activated by phosphorylation and PXN binds to CRKL once phosphorylated. Therefore, inhibitors of tyrosine kinases can lead to the down-regulation of CRKL and PXN, a fact that is being now taken into consideration for the development of novel RA drugs [37]. Indeed, a recent clinical trial (phase II) shows promising results in the control of a specific tyrosine [38], based on clinical efficacy observed in mice where the small molecule R406 is used to target the spleen tyrosine kinase (Syk), which plays a crucial role in the signalling of activating Fc receptors and the B-cell receptor [39]. From our network analysis in human, we can suggest that the control of synovium degeneration illustrated above might be an additional reason to its efficacy, as Syk is directly linked to CRKL [40,41]. Additionally, we can suggest an interpretation to one of the side effects of the therapy: neutropenia. Due to the crucial role of CRKL in neutrophil adhesion (control of neutrophils’ spatial activation [35]), CRKL indirect down-regulation via Syk for the control of synovium degeneration implies a reduction in neutrophils activity.

The control (down-regulation) of PXN was also proposed as a novel therapeutic approach for RA, as PXN is up-regulated in this disease via the triggering of the FAK family kinases signalling cascade [42]. To our knowledge this path has not (yet) been pursued. In general, the control of PXN can be achieved through a hierarchy of interactions and therefore this represents a more subtle system that cannot be targeted by the ‘golden bullet’ therapy approach. Interestingly *insulin*, via the tyrosine dephosphorylation of PXN, is able to control (reduce) its activity in conjunction with Phosphotyrosyne Phosphatase 1D [43]. Phosphotyrosyne Phosphatase 1D is encoded by *PTPN11* and controlled (inhibited) by echistatin [44]. This is extremely significant as a large amount of literature exists on insulin related to diabetes. The overlap between RA and diabetes is an interesting one: in RA and diabetes, levels of inflammatory markers, such as C-reactive protein (CRP), *TNF*α and interleukin-6 (*IL-6*) are typically increased and drugs used to treat RA by reducing inflammation -through inhibition of *TNF*α- have shown promising results in the treatment of diabetes [45]. Reducing inflammation with *Remicade* has also shown to improve insulin sensitivity in people who had inflammatory diseases and were insulin resistant [46]. It therefore follows suite that some treatments for one inflammatory disease may be effective in the treatment of the other. In this direction, an on-going clinical study (NCT00763139) is testing Pioglitazone (commonly used for diabetes treatment) on RA. This trial is based on the rationale that recent studies have shown an increased prevalence of coronary artery atherosclerosis, metabolic syndrome and insulin resistance among people with RA. Furthermore, insulin resistance, which can lead to hyperinsulinemia -too much insulin in the blood- has been associated with RA disease activity and the severity of coronary artery atherosclerosis. These correlations corroborate the observation that inflammation and hyperinsulinemia somehow interact and facilitate one another. Interestingly, by joining all the above information, thanks also to the systemic view that the map allowed us to have, we can recommend not to use Pioglitazone in conjunction with echistatin and therefore add this interaction as an unsuitable one. This is a non-trivial result of our simulation as echistatin has already been suggested as a possible compound for the treatment of RA [47].

## Conclusions

The regulation of CRKL represents a typical systems biology problem, such that in order to justify using experimental techniques to understand the mechanisms in which the molecule(s) of interest is (are) involved, sound hypotheses need to be formulated. The computational methods adopted here have been specifically designed to simulate a dynamic element in a previous static representation of a biological network, providing essential support for guiding future research, whilst also allowing for a systemic view of the biological network of interest.

The results of the simulation modelling also draw our attention to several genes known to be involved in RA and, specifically, the triggering of the RAS and JUN pathways; typical phenomena occurring in the degenerate synovium, characterizing the condition [21]. As CRKL appears to be up-regulated in RA, we hypothesise that the *in silico* analysis [12] that suggests CRKL as a possible target for the RA therapy reflects the biological rationale that the down-regulation of CRKL -which controls the activation of PXN- represents a mean to control the synovium degeneration.

Based on these observations, our approach has allowed us, importantly, to move one step forward i.e. to use the results of simulations to suggest practical and meaningful information to be translated in clinical practice.

The results obtained thus have allowed us to generate more refined hypothesis *in silico* that have the potential to advise and impact the future of RA research.

## Competing interests

The authors declare that they have no competing interests.

## Authors' contributions

JED and CN designed the study. JED did the network analysis. CN did the biological interpretation of results. JED and CN wrote the manuscript. Both authors read and approved the final manuscript.

## Supplementary Material

Additional File 1 **Molecular-interaction map for RA.** (a) protein-protein interaction map, (b) gene regulation map. The two maps are joined by transcription factors. For a more comprehensive view, we recommend to visualize the figure with CellDesigner [22]. Click here for file

Additional File 2 **CRKL sub-network.** Edges correspond to SBML reaction types: black - modifier; green - product; red - reactants. Reactions are labelled by a circle and species are labelled by a diamond. Click here for file

Additional File 3 **Mean expression level of network molecules for CRKL down-regulated.** Simulated change in expression levels of a sample of molecules connected to CRKL, for low starting levels of CRKL (CRKL excluded). Sample chosen to display every 10th molecule, sorted by expression level at time t = 20. Click here for file

Additional File 4 **Mean expression level of network molecules for CRKL up-regulated.** Simulated change in expression levels of a sample of molecules connected to CRKL, for high starting levels of CRKL (CRKL excluded). Sample chosen to display every 10th molecule, sorted by expression level at time t = 20. Click here for file

Additional File 5 **Mean expression level of network molecules significantly affected by regulation of CRKL. a)** Simulated change in expression levels of molecules connected to CRKL, for CRKL down-regulated and **b)** Simulated change in expression levels of molecules connected to CRKL, for CRKL up-regulated. Nodes correspond to those in Table 1. Click here for file

Additional File 6 **Summary table of molecules.** Summary of aliases, function and ontology (as described by GeneCard [31]) for molecules in Table 1. Click here for file
