# Supplementary figures and images for: From desk to bed: Computational simulations provide indication for rheumatoid arthritis clinical trials
Source: BMC Syst Biol. 2013 Jan 22;7:10. doi: 10.1186/1752-0509-7-10 (PMC3653749; doi:10.1186/1752-0509-7-10)

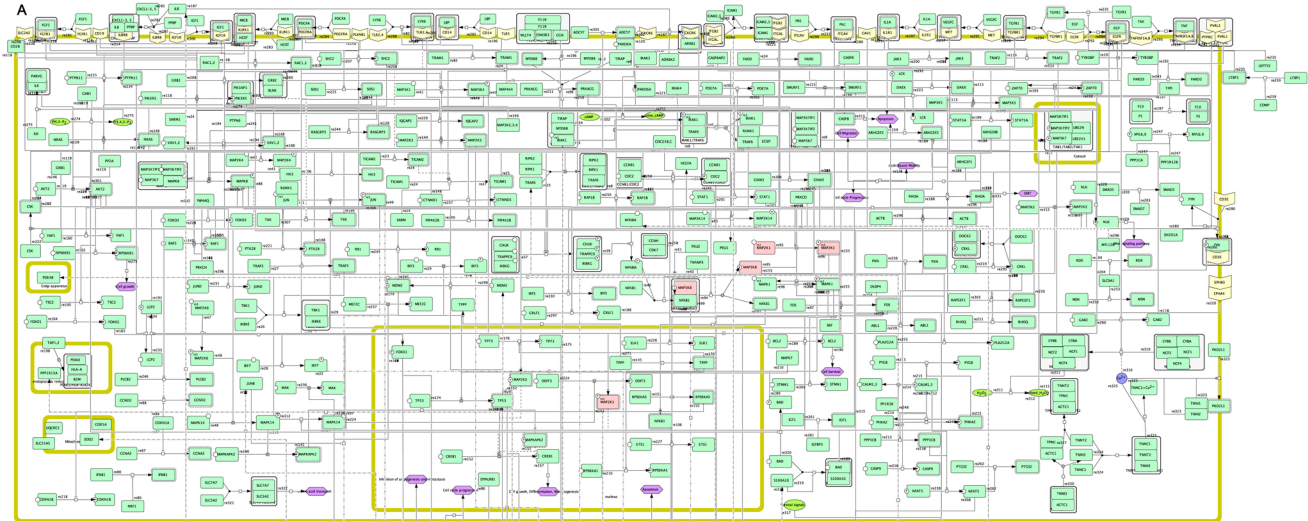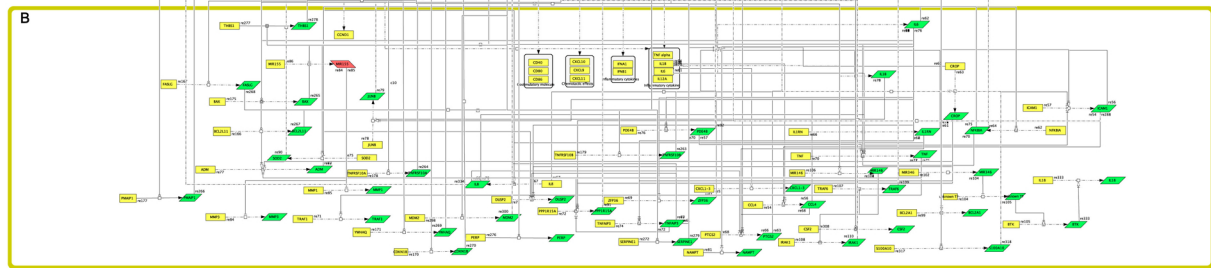

Supplement: Additional File 1 — Molecular-interaction map for RA. (a) protein-protein interaction map, (b) gene regulation map. The two maps are joined by transcription factors. For a more comprehensive view, we recommend to visualize the figure with CellDesigner [22]. [file 1752-0509-7-10-S1.pdf]

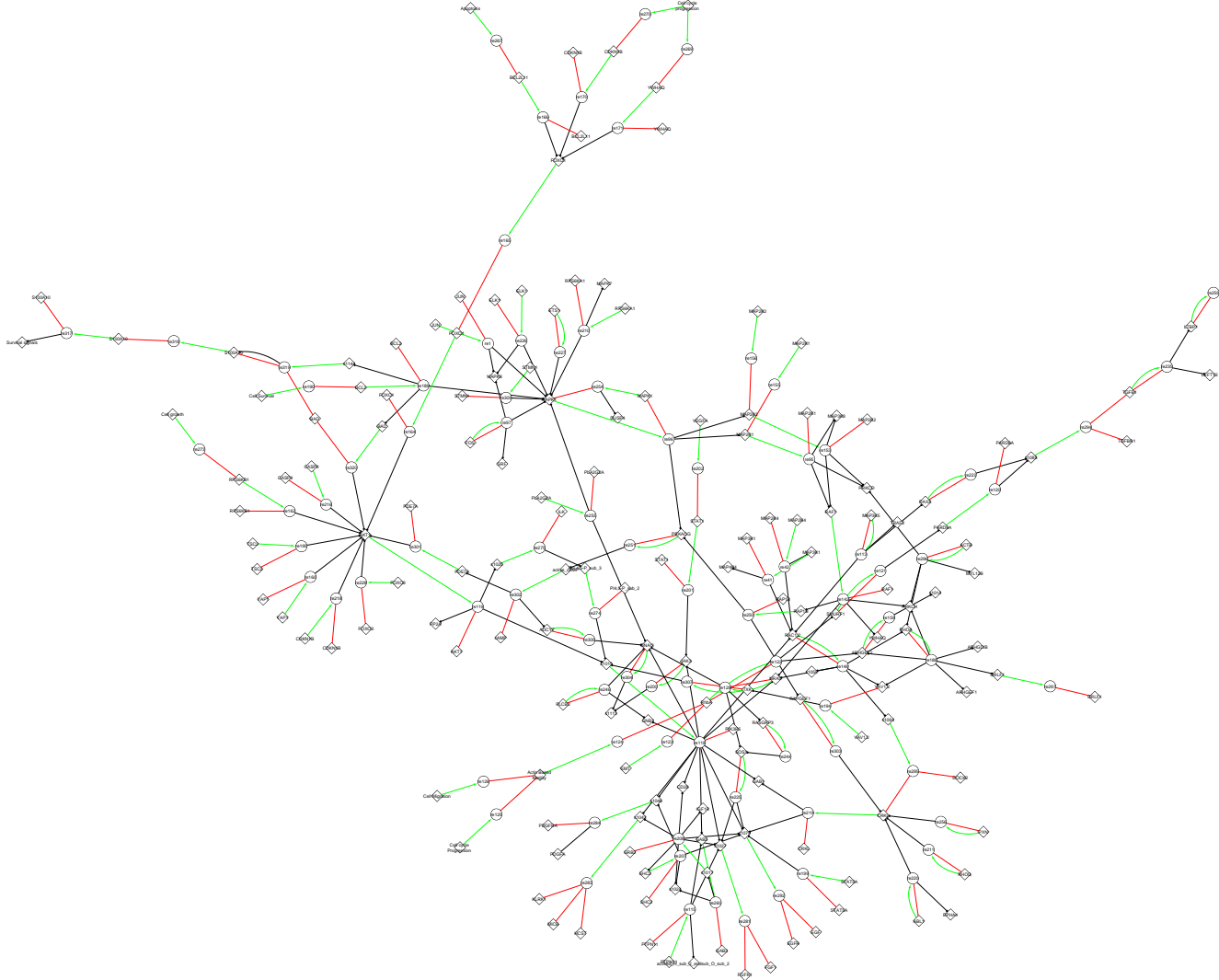

Supplement: Additional File 2 — CRKL sub-network. Edges correspond to SBML reaction types: black - modifier; green - product; red - reactants. Reactions are labelled by a circle and species are labelled by a diamond. [file 1752-0509-7-10-S2.pdf]

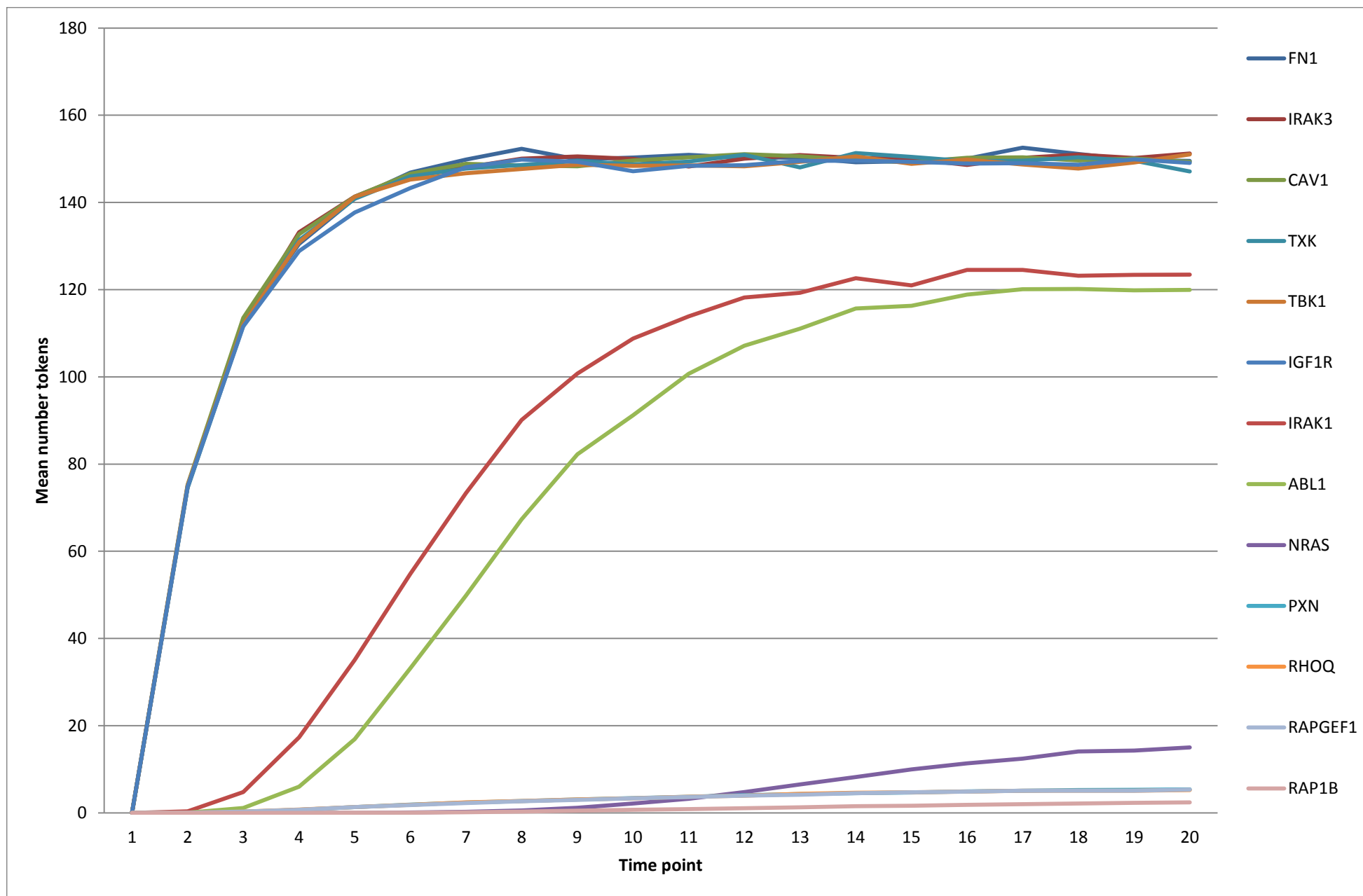

Supplement: Additional File 3 — Mean expression level of network molecules for CRKL down-regulated. Simulated change in expression levels of a sample of molecules connected to CRKL, for low starting levels of CRKL (CRKL excluded). Sample chosen to display every 10th molecule, sorted by expression level at time t = 20. [file 1752-0509-7-10-S3.pdf]

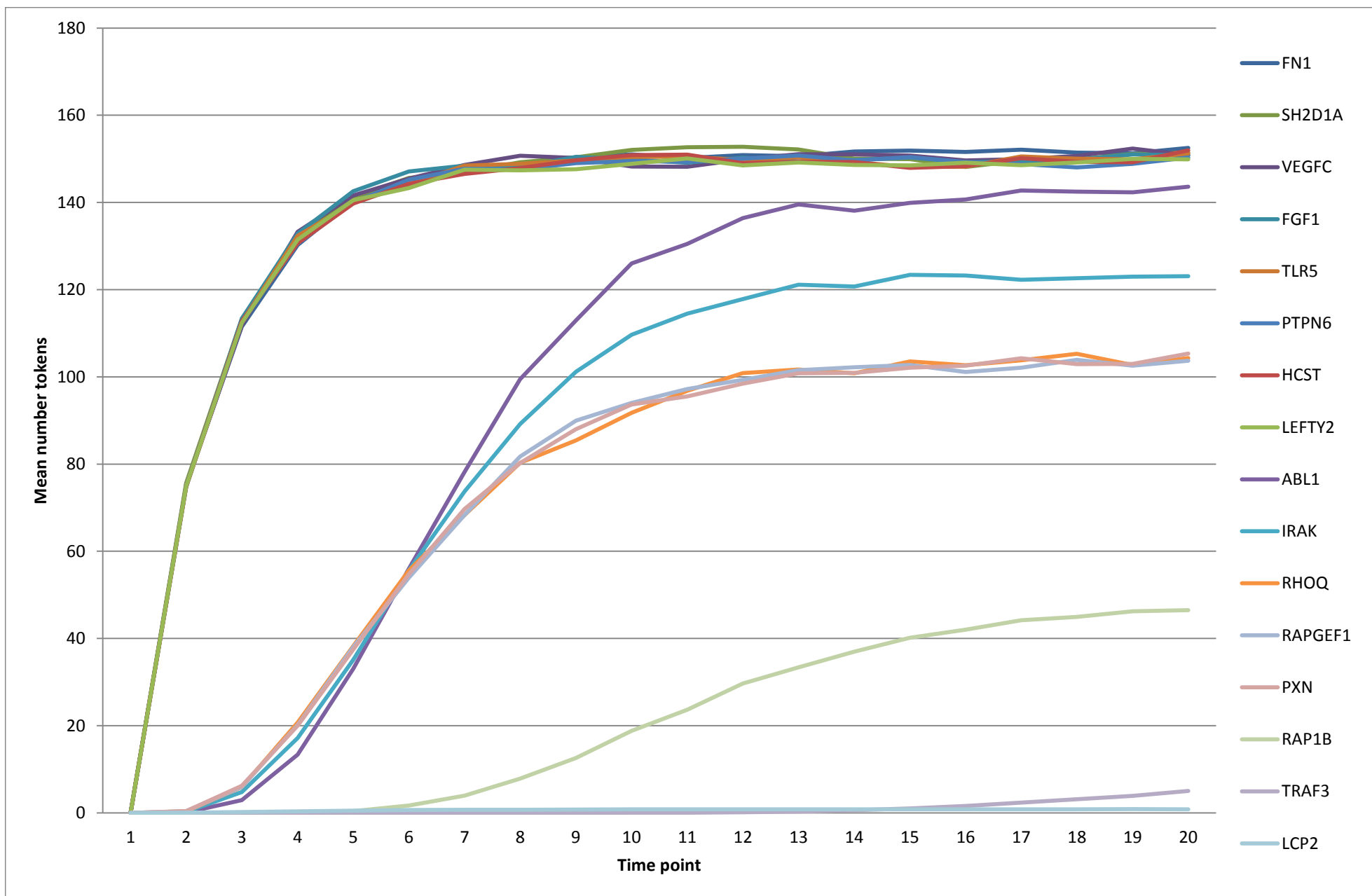

Supplement: Additional File 4 — Mean expression level of network molecules for CRKL up-regulated. Simulated change in expression levels of a sample of molecules connected to CRKL, for high starting levels of CRKL (CRKL excluded). Sample chosen to display every 10th molecule, sorted by expression level at time t = 20. [file 1752-0509-7-10-S4.pdf]

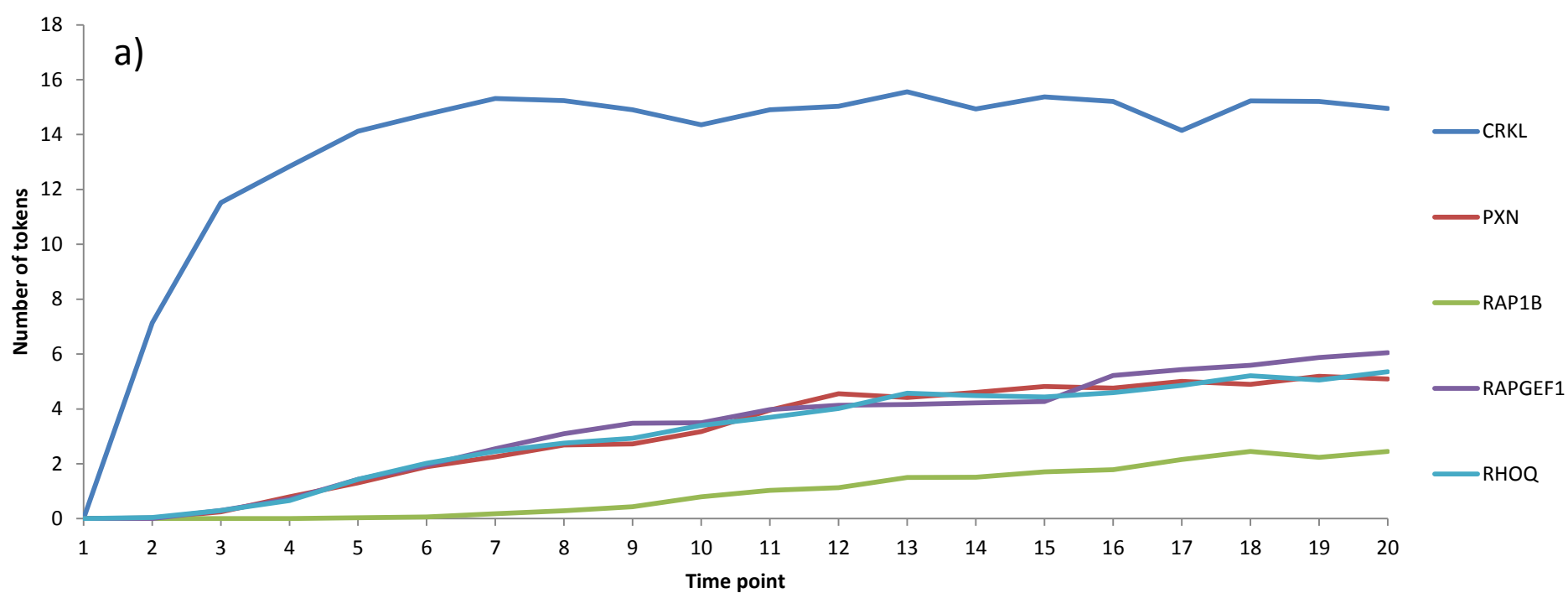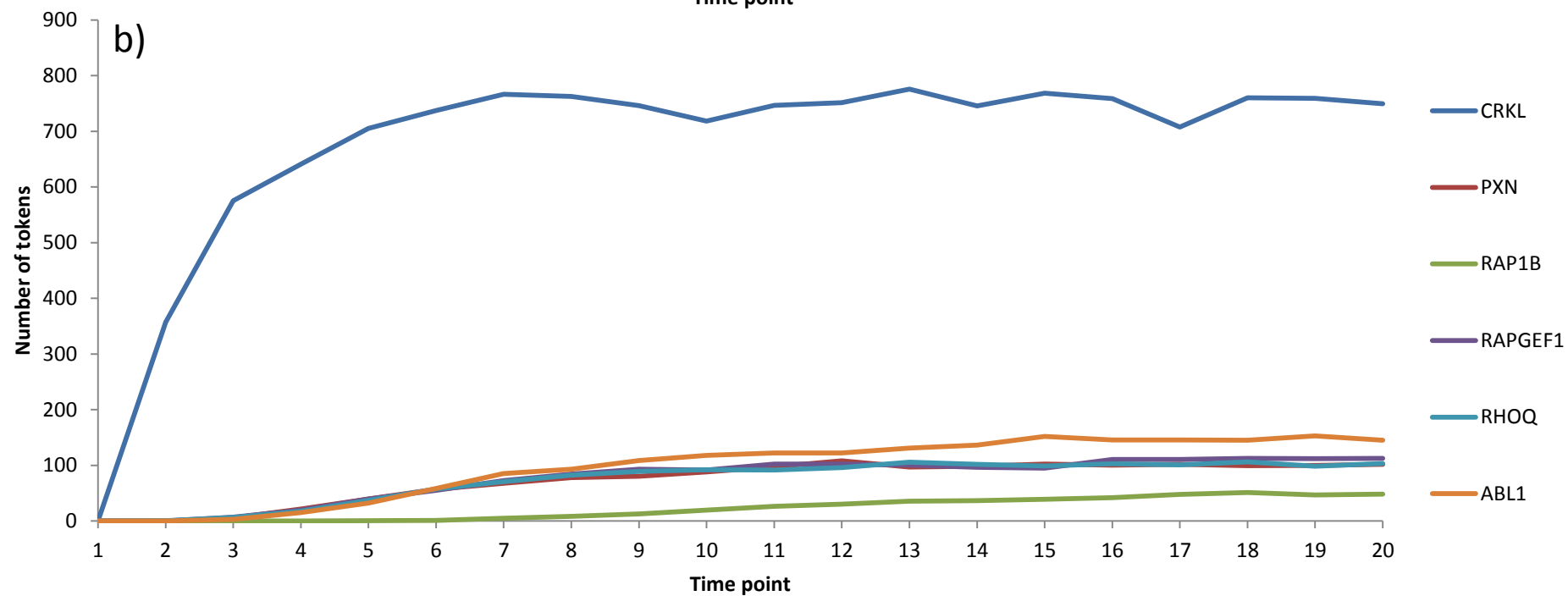

Supplement: Additional File 5 — Mean expression level of network molecules significantly affected by regulation of CRKL. a) Simulated change in expression levels of molecules connected to CRKL, for CRKL down-regulated and b) Simulated change in expression levels of molecules connected to CRKL, for CRKL up-regulated. Nodes correspond to those in Table 1. [file 1752-0509-7-10-S5.pdf]
